# An efficient procedure for protein extraction from formalin-fixed, paraffin-embedded tissues for reverse phase protein arrays

**DOI:** 10.1186/1477-5956-10-56

**Published:** 2012-09-24

**Authors:** Huifang Guo, Wenbin Liu, Zhenlin Ju, Pheroze Tamboli, Eric Jonasch, Gordon B Mills, Yiling Lu, Bryan T Hennessy, Dimitra Tsavachidou

**Affiliations:** 1 Department of Systems Biology, University of Texas MD Anderson Cancer Center, Houston, TX, USA; 2 Department of Bioinformatics and Computational Biology, University of Texas MD Anderson Cancer Center, Houston, TX, USA; 3 Department of Pathology, University of Texas MD Anderson Cancer Center, Houston, TX, USA; 4 Department of Genitourinary Medical Oncology, University of Texas MD Anderson Cancer Center, Houston, TX, USA; 5 Department of Medical Oncology, Beaumont Hospital, Royal College of Surgeons of Ireland, Dublin, Ireland

**Keywords:** Formalin-fixed, Paraffin-embedded tissue, Protein extraction, Reverse phase protein array, Breast cancer, Renal cancer

## Abstract

**Introduction:**

Protein extraction from formalin-fixed paraffin-embedded (FFPE) tissues is challenging due to extensive molecular crosslinking that occurs upon formalin fixation. Reverse-phase protein array (RPPA) is a high-throughput technology, which can detect changes in protein levels and protein functionality in numerous tissue and cell sources. It has been used to evaluate protein expression mainly in frozen preparations or FFPE-based studies of limited scope. Reproducibility and reliability of the technique in FFPE samples has not yet been demonstrated extensively. We developed and optimized an efficient and reproducible procedure for extraction of proteins from FFPE cells and xenografts, and then applied the method to FFPE patient tissues and evaluated its performance on RPPA.

**Results:**

Fresh frozen and FFPE preparations from cell lines, xenografts and breast cancer and renal tissues were included in the study. Serial FFPE cell or xenograft sections were deparaffinized and extracted by six different protein extraction protocols. The yield and level of protein degradation were evaluated by SDS-PAGE and Western Blots. The most efficient protocol was used to prepare protein lysates from breast cancer and renal tissues, which were subsequently subjected to RPPA. Reproducibility was evaluated and Spearman correlation was calculated between matching fresh frozen and FFPE samples.

The most effective approach from six protein extraction protocols tested enabled efficient extraction of immunoreactive protein from cell line, breast cancer and renal tissue sample sets. 85% of the total of 169 markers tested on RPPA demonstrated significant correlation between FFPE and frozen preparations (p < 0.05) in at least one cell or tissue type, with only 23 markers common in all three sample sets. In addition, FFPE preparations yielded biologically meaningful observations related to pathway signaling status in cell lines, and classification of renal tissues.

**Conclusions:**

With optimized protein extraction methods, FFPE tissues can be a valuable source in generating reproducible and biologically relevant proteomic profiles using RPPA, with specific marker performance varying according to tissue type.

## Background

Formalin fixation coupled with paraffin embedding (FFPE) is the standard tissue fixation and storage method adopted by most health institutions. FFPE tissues are highly stable, and can be stored at room temperature. Proteins are stabilized through cross-linking and cell or tissue structure is mainly preserved. Multiple reports show that proteins and protein modifications such as phosphorylation are maintained and can be determined years later by immunohistochemistry [1].

However, releasing proteins from FFPE tissues for proteomic analysis has proven to be a daunting task. The extensive molecular crosslinking that occurs upon formalin fixation reduces protein extraction efficiency and may interfere with immunoreactivity [2], warranting the development of more efficient extraction methods [3]. Several proteomic studies using archival FFPE tissues have been reported in recent years. The majority of these studies employ protein extraction methods that are derived from heat-induced antigen retrieval techniques originally developed for immunohistochemistry [4-8].

In the era of personalized medicine, new therapeutic and diagnostic options are rapidly becoming available to cancer patients, based on novel high-throughput technologies. Reverse-phase protein array (RPPA) can efficiently quantify changes in protein levels and protein functionality in numerous tissue and cell sources [9-12]. This methodology provides a suitable approach in generating high-throughput protein profiles in a robust quantitative manner, providing an advantage over traditional methods such as immunohistochemistry [13,14]. RPPA has been used to evaluate protein expression mainly in frozen preparations. Reproducibility and reliability of the technique in FFPE samples have not been previously assessed extensively.

In this study, we developed and optimized an efficient and reproducible procedure for extraction of proteins from FFPE tissues. We demonstrate that this approach produces reliable results and biologically meaningful proteomic profiles generated by RPPA.

## Results

### Optimization of the protein extraction method from paraffin-embedded samples

Paraffin-embedded specimens comprise a wide variety of tissues, which may include samples of very limited amount, such as core biopsies. Protein material extracted from paraffin-embedded specimens may be of lower quality compared to frozen equivalents, because of degradation or dephosphorylation of proteins due to the formalin fixation and embedding process or differences in time taken for preparation. It is therefore important to evaluate protein extraction methods for optimal performance, both in terms of quantity as well as quality.

FFPE blocks generated from breast cancer cell lines and xenografts were used for preliminary studies due to ready availability and the ability to control conditions of preparation and handling. We applied different protein extraction protocols to cell lines and xenografts embedded in paraffin, in order to evaluate yield and protein quality. The protocols used in this study were versions of previously described RPPA protocols [9,15], modified to include deparaffinization steps. Additional modifications were adopted based on an extensive literature review [16-18]. A commercially available protocol (Qproteome FFPE Tissue Kit from Qiagen, Germany) was also assessed. In particular, method 1 was a modification of the RPPA extraction protocol [9,15] to include deparaffinization steps. Methods 2 and 3 are versions of method 1 based on literature [16]. Methods 4–6 are based on method 2 with buffers of varying pH (described in Table 1).

**Table 1:** Protein extraction methods

	**Method 1**	**Method 2**	**Method 3**	**Method 4**	**Method 5**	**Method 6**	**Qiagen**
**RPPA buffer**	✓	✓	✓				
**Qproteome buffer**							✓
**20mM Tris–HCl, pH=9**				✓			
**20mM Tris–HCl, pH=7.4**					✓		
**20mM Tris–HCl, pH=4**						✓	
**sonication**	✓		✓				
**incubation at 80**^**o**^**C**		✓	✓	✓	✓	✓	

The differences in buffer type and conditions between the protocols for protein extraction from FFPE cells and tissues used in the study are shown.

**Figure 1:**
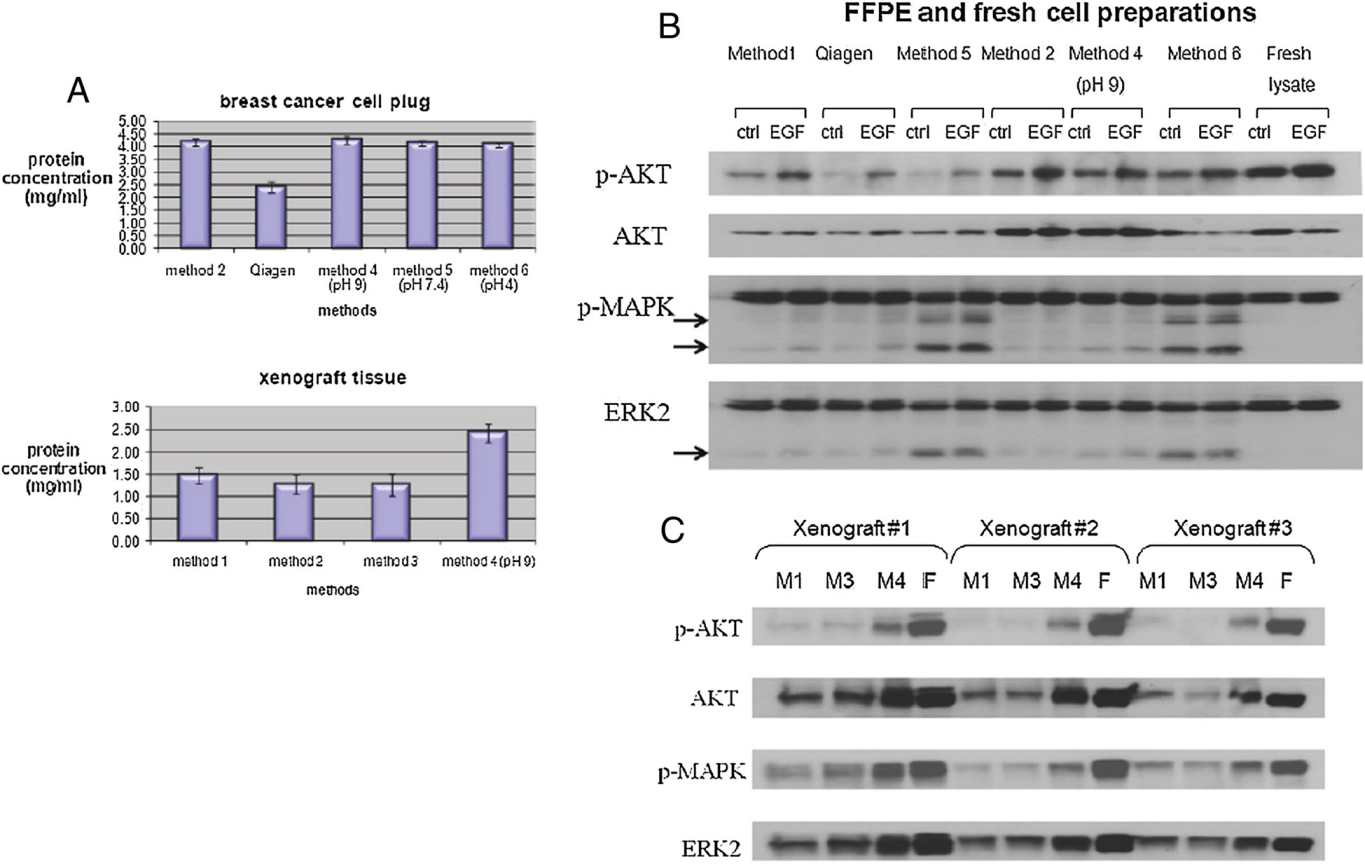
**Protein yield and signal quality using modified extraction methods.** 1**A**: Protein concentration of lysates generated from breast cancer cell FFPE plugs (upper panel) and FFPE xenografts (lower panel) using different extraction methods is shown. 1**B**: Western blots for select protein markers are shown. Extracts from breast cancer cell lines incubated with or without EGF (marked “EGF” and “ctrl” respectively) were derived from FFPE or fresh preparations using different extraction methods and subjected to western blotting. 1**C**: Western blots using lysates from xenografts are shown. M1 = method 1; M3 = method 3; M4 = method 4; F = lysate from frozen preparation. Arrows point to low molecular weight bands attributed to protein degradation.

We first tested the protein concentration of the extracts recovered in standard volume, using the various methods, for both cell and xenograft preparations. Method 4 (the modification of method 2, with pH = 9) showed the highest yield (protein concentration of 4.25 mg/ml in breast cancer cell FFPE preparations, and 2.43 mg/ml in xenograft FFPE preparations) compared to the other methods (Figure 1A). We then evaluated the degree of degradation of the extracted protein. Specifically, we ran extracts from paraffin-embedded cell lines and xenograft tissues on SDS-PAGE gels, in parallel with extracted protein material from frozen preparations, stained the gels with Coomassie blue, and assessed protein degradation as evidenced by the presence of low molecular weight bands or loss of signal. Coomassie blue staining demonstrated that the extracts from paraffin-embedded specimens (cell plugs and xenograft tissues) showed some prominent lower weight products (Additional file 6: Figure S2A, B), but with recoverable protein at high molecular weights as well. In addition to degradation, differences in lysis buffers may contribute to the variability in the observed banding patterns. Western blot analysis for select signaling molecules confirmed that there was some protein degradation in the FFPE samples, which was reflected by lower molecular weight bands, but the bands of the correct molecular weight were strong, as in the case of frozen samples (Figure 1B, C). Importantly, the phosphorylation status of the proteins tested was conserved, as evidenced by phospho-AKT and phospho-ERK levels comparable between FFPE and frozen preparations.

### RPPA on paraffin-embedded specimens

After establishing that method 4 exhibited the highest protein yields and showed only partial protein degradation on Coomassie staining and western blots, we proceeded with RPPA, in order to evaluate protein expression in extracts processed with method 4. In particular, extracts derived from paraffin-embedded cell line samples and their matched fresh frozen preparations were spotted on arrays and stained for 169 protein markers. Each fresh frozen (FF) and FFPE sample was represented by two replicate preparations on the array. Each replicate preparation was serially diluted and spotted as described in Methods and Additional file 5: Figure S1. The resulting data were normalized using GSMN (see Methods). A breast and ovarian cancer cell line panel of six lines grown under different cell culture conditions (serum-starved media, addition of growth factors (EGF or IGF) or pharmacological inhibitors such as PI3K inhibitor LY294002 and MAPK inhibitor PD98059) provided a dynamic range for phosphorylation events.

**Figure 2:**
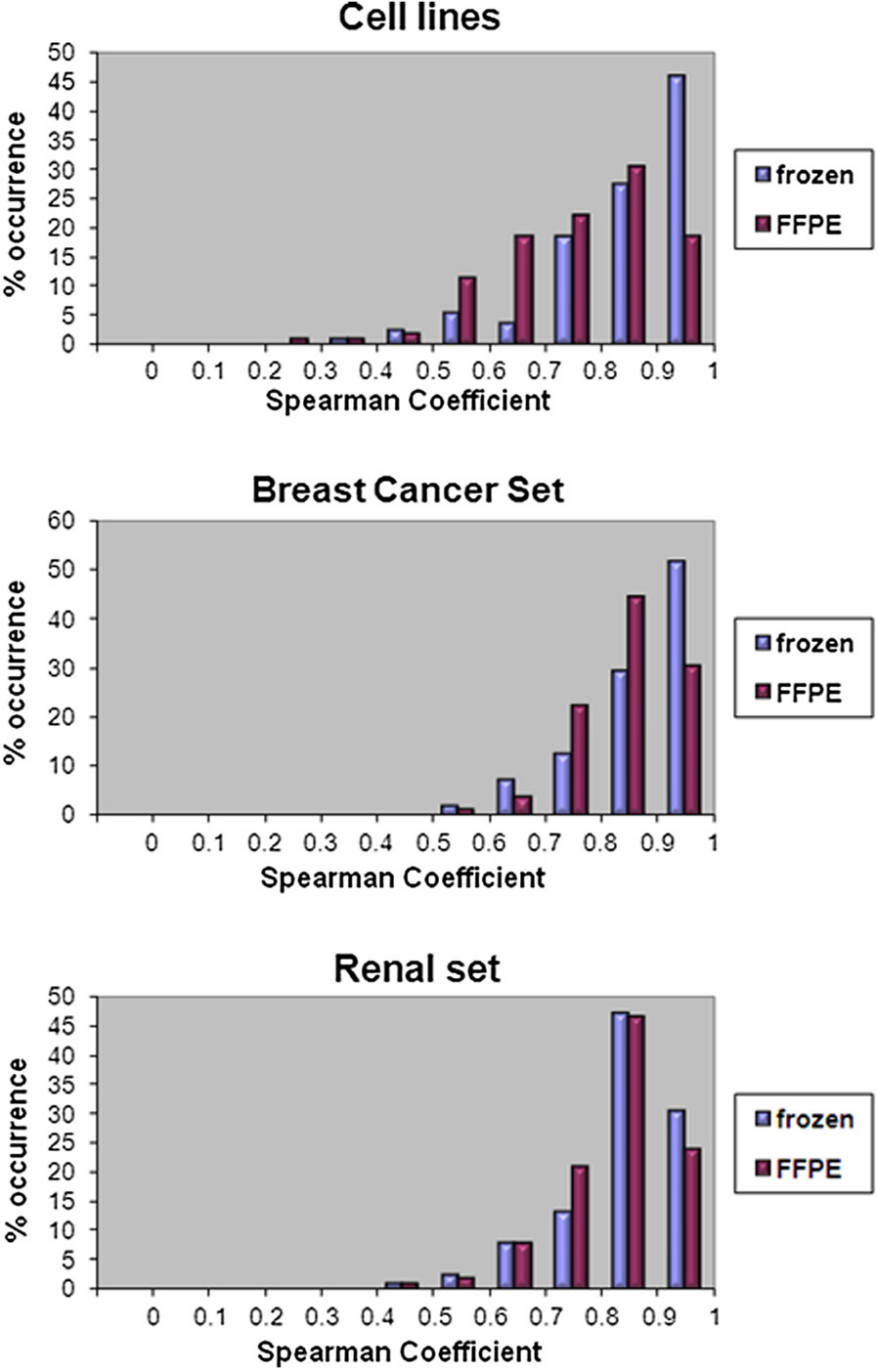
**RPPA reproducibility between replicate preparations.** Reproducibility between replicate preparations is expressed as the percentage of markers (% occurrence) at distinct intervals of Spearman coefficient.

The purpose of this experiment was to evaluate RPPA performance on paraffin-embedded material in a highly controlled and predictable experimental setting. We first addressed reproducibility of the technique by estimating the Spearman correlation coefficient between replicate preparations across the protein markers tested. Both formalin fixed paraffin-embedded (FFPE) and frozen replicates performed reproducibly. Specifically, 91.42% of markers showed reproducibility between frozen sample replicates with spearman coefficient equal or greater than 0.7 (p < 3.5*10^-7^). For the FFPE sample replicates, 70.41% of markers showed reproducible results (spearman coefficient > =0.7, p < 1.48*10^-6^) (Figure 2).

In addition to technical reproducibility, we assessed how similar the FFPE preparations performed compared to matched fresh frozen specimens. After estimating the Spearman coefficient between FFPE and matched FF samples across all protein markers in the RPPA experiment, we observed that there was statistically significant correlation (cutoff p <0.05, spearman coefficient > 0.36) for 45% of the markers tested (77 markers) (Table 2). For the markers that did not demonstrate significant correlation between FFPE and their matched frozen preparations, we sought to investigate potential reasons for the apparent lack of correlation. First, we considered the case of overall low signal. Indeed, 17% of the protein markers exhibited low intensities (log2 intensities of < −2), for all the samples tested. We then evaluated whether large intensity differences between the FFPE and FF sample sets may account for the observed low correlation. 32% of all the protein markers had significantly different signal intensities (> 2-fold, p < 5*10^-6^) between the FFPE and FF sets and low correlation between FFPE and FF intensities (Table 2). In fact, there was a significant enrichment of markers with different signal intensities between FFPE and FF in the low correlation group (59% of markers with low correlation between FFPE and FF exhibited high intensity differences between the two preparations, whereas only 23% of markers with good correlation between FFPE and FF had high intensity differences). There was no apparent bias towards a specific preparation type, with overall low or overall high intensities being equally distributed between the FFPE and FF sets.

**Table 2:** Distribution of RPPA-detected protein markers depending on their performance between frozen and FFPE preparations

	**Markers with p < 0.05 between FFPE and FF**	**Markers with large intensity difference between FFPE and FF**	**Markers with low intensity in both FFPE and FF**	**Markers with low replicate reproducibility (R < 0.7)**
**Cell lines**	77 (45%)	54 (32%)	28 (17%)	10 (6%)
**Breast cancer set**	54 (32%)	81 (48%)	25 (15%)	9 (5%)
**Renal cancer set**	107 (64%)	31 (18%)	7 (4%)	24 (14%)

Markers with high correlation (p < 0.05) between frozen and FFPE preparations, large intensity differences, low signal or low reproducibility for cell lines, breast cancer set and renal set are shown.

The remaining 6% of markers (Table 2) with poor correlation between FFPE and FF were found to have poor reproducibility between replicates for either FFPE or FF preparations, due to yet undetermined reasons.

**Figure 3:**
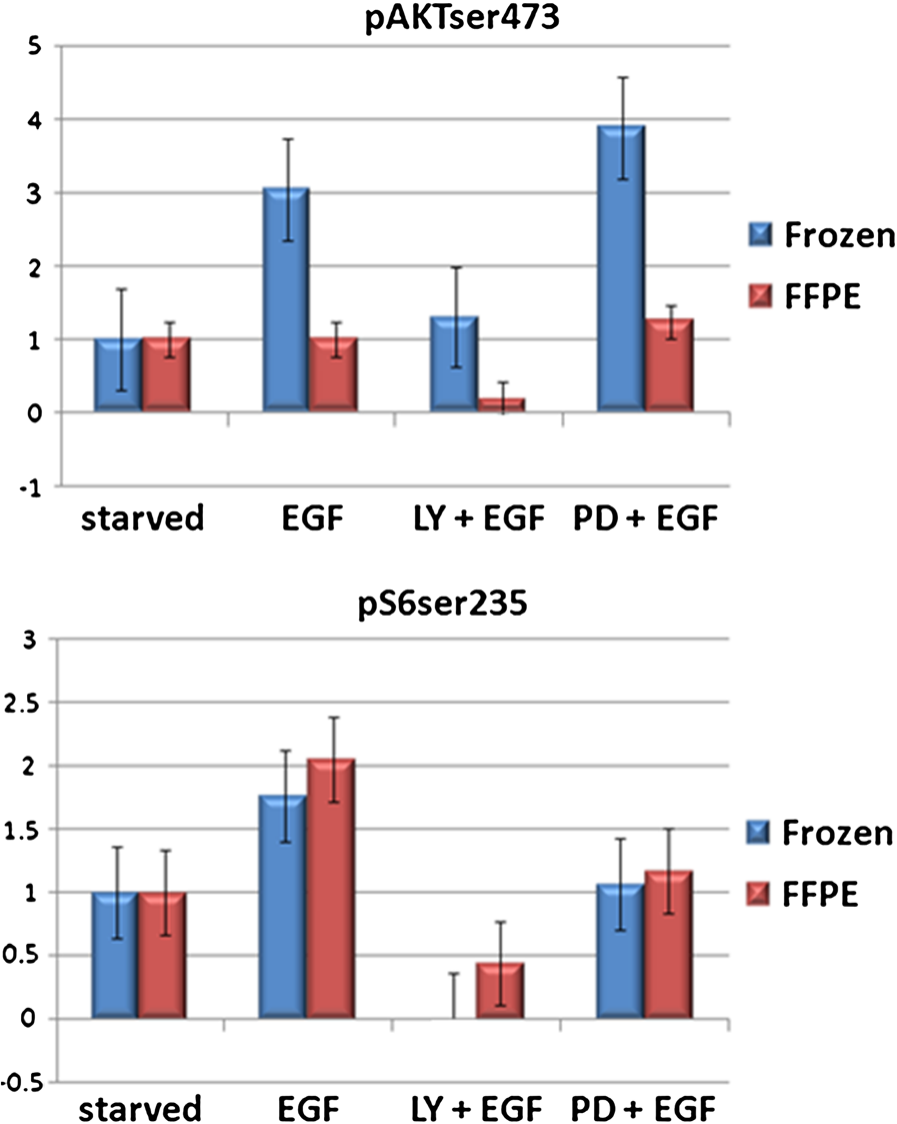
**Phosphorylation patterns of the AKT pathway according to cell treatment and preparation type.** Phospho-AKT and phospho-S6 were detected using RPPA. The lysates spotted on the protein arrays were derived from breast and ovarian cancer cell lines treated with growth factors or inhibitors. Starved = serum-starved cell cultures; EGF = growth factor treated; LY = LY294002; PD = PD98059.

To test whether results derived from FFPE specimens could be used to identify biologically relevant changes in signaling events, we focused on phospho protein markers, where levels were altered by changing culture conditions and applying pharmacological interventions. In particular, we expected phospho-AKT (pAKTser473) and its downstream effector phospho-S6 (pS6ser235) to be induced by growth factor addition in the culture media, and inhibited by PI3K inhibitor (LY294002). On the other hand, these markers should not be affected directly by a MAPK inhibitor (PD98059). Indeed, we observed that both phospho-AKT and phospho-S6 exhibited the anticipated changes in terms of rank order in matched FFPE and frozen samples (Figure 3). Specifically, the average normalized log2 signal intensities of both pAKTser473 and pS6ser235 increased by 3-fold and 1.76-fold respectively in the epidermal growth factor (EGF)-induced cultures in frozen preparations. pS6ser235 was increased by 2.05-fold in EGF-treated FFPE preparation, similar to that in the EGF-treated frozen preparation, indicating that the pS6ser235 site was robust to handling. In contrast, EGF-induced elevation in pAKTser473 was not detected in the FFPE preparation, suggesting that pAKT was dephosphorylated during handling. Compared to EGF-treated cultures, pAKTser473 and pS6ser235 levels were markedly reduced in the LY294002-treated cultures, as anticipated (pAKTser473; 2.3-fold reduction in frozen and 5.2-fold reduction in FFPE preparations, pS6ser235; >10-fold reduction in frozen and 4.66-fold reduction in FFPE). PD98059 treatment did not substantially alter the effects of EGF on pAKTser473 and pS6ser235 in either FFPE or frozen preparations (Figure 3).

The experiment on cell lines provided evidence supportive of applicability of high-throughput protein expression detection from FFPE specimens. We then sought to analyze RRPA-based protein expression patterns for a set of FFPE breast cancer tissues from human patients. In particular, we screened 49 breast cancer samples along with an equal number of replicate preparations and matched frozen samples for expression of 169 protein markers. The replicates from the same specimen performed similarly for both FFPE and frozen preparations (Figure 2). Specifically, 92.3% of markers showed reproducibility between frozen sample replicates with spearman coefficient equal or greater than 0.7 (p < 5*10^-10^). For the FFPE sample replicates, 96.4% of markers showed reproducible results (spearman coefficient > =0.7, p < 6.35*10^-10^). When the FFPE data were compared to their frozen counterparts, 32% demonstrated similar expression patterns (cutoff p <0.05, spearman coefficient > 0.28) between FFPE and matched frozen samples (Table 2). Importantly, 48% of the markers exhibited statistically significant intensity differences (> 2-fold, p < 5*10^-6^) between frozen and FFPE preparations, implying that a number of processes affecting protein integrity such as handling, formalin fixation, protein extraction buffers and conditions, as well as tissue heterogeneity may contribute to differences between FFPE and matched frozen samples. Nevertheless for those proteins demonstrating correlations, FFPE may provide a usable source of material.

**Figure 4:**
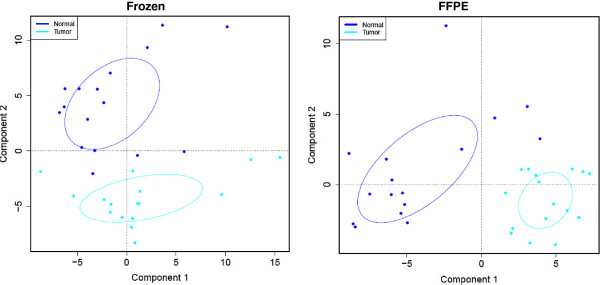
**Principal component analysis (PCA) of protein expression profiles of the renal set.** Protein expression profiles depicted as PCA plots were generated using lysates from frozen (left) or FFPE preparations (right) spotted on protein arrays using the RPPA approach. The ellipses were centered based on the means of the first and second principal components for each group (normal or tumor), and drawn using the variation factors that are the products of the eigenvector and the square root of eigenvalues calculated from the variances and covariances of the first and second principal components. The two groups are separated, as indicated by their means.

We also assessed two additional tissue types, renal cancer and normal renal cortex. These specimens were paraffin-embedded following a method similar to the one employed in the cell line experiment. We processed 15 renal cancer samples and 16 normal renal cortex samples from human patients. Each sample was represented by two replicate frozen and two replicate FFPE preparations. Similarly to our previous experiments, we established technical reproducibility. Specifically, 89.94% of markers showed reproducibility between frozen sample replicates with spearman coefficient equal or greater than 0.7 (p < 2.5*10^-7^). For the FFPE sample replicates, 90.53% of markers showed reproducible results (spearman coefficient > =0.7, p < 3.66*10^-7^) (Figure 2). Correlation between FFPE and frozen preparations was improved compared to the cell line and breast cancer experiments, with 64% of the markers exhibiting p < 0.05 (spearman coefficient > 0.35)(Table 2). Importantly, the RPPA-based global protein expression profile of the FFPE preparations was able to distinguish between normal renal and tumor samples, as evidenced by means of principal component analysis (Figure 4).

**Figure 5:**
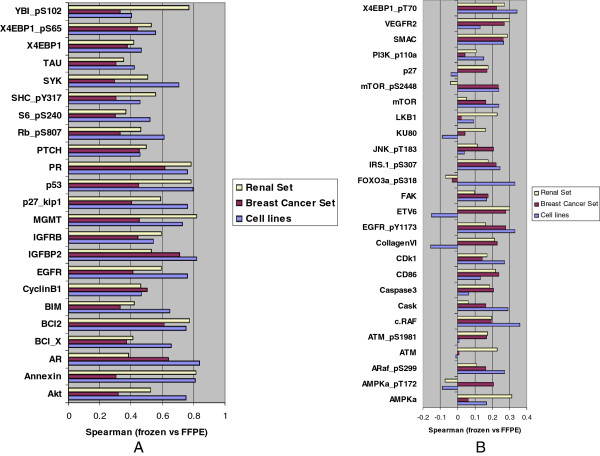
**Protein markers and their correlation between FFPE and frozen preparations. A**: Markers with high correlation (p < 0.05) between frozen and FFPE preparations in all of the studied cell and tissue sets are shown. **B**: Markers with poor correlation (p >0.05) for all three sets are shown.

Comparing marker performance across all three experiments, the majority of protein markers (85%) demonstrated significant correlation between FFPE and frozen preparations (p < 0.05) in at least one experiment (cell lines, breast cancer set or renal set), with 23 markers exhibiting significant correlation in all three data sets (Figure 5A). There was variability between breast, renal and cell line data for each antigen suggesting that tissue specific characteristics likely contribute to total or phospho protein stability or recovery. The 26 markers with poor performance as evidenced by low frozen vs. FFPE correlations are shown in Figure 5B.

**Figure 6:**
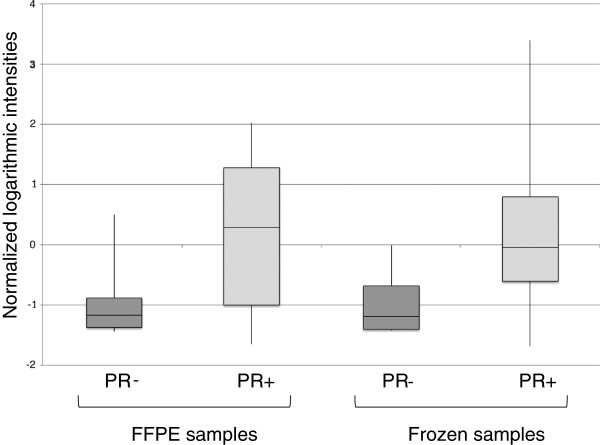
**RPPA-generated protein expression levels of PR according to clinical PR status.** Normalized logarithmic intensities corresponding to RPPA-generated protein expression levels of PR in FFPE and matched frozen breast tumor samples are shown, grouped according to immunohistochemistry-based clinical status of PR (PR - = PR-negative; PR + = PR-positive status).

In order to demonstrate the applicability of RPPA from FFPE materials in a clinical laboratory setting, we sought to validate our results by comparing them to available immunohistochemistry-generated data of clinically significant markers. In particular, we compared our breast cancer RPPA data for one of our best performing markers (Figure 5A), PR (progesterone receptor), with corresponding immunohistochemical measurements. As shown in Figure 6, the breast cancer samples characterized as PR-positive using immunohistochemistry showed significantly higher PR protein expression levels in RPPA, compared to the PR-negative tumors. This was the case for both FFPE (PR-negative median normalized logarithmic intensity = −1.19; PR-positive median normalized logarithmic intensity = 0.38; p < 0.0008) and frozen samples (PR-negative median normalized logarithmic intensity = −1.19; PR-positive median normalized logarithmic intensity = −0.06; p < 2.5*10^-5^).

## Discussion

In this study, we have shown that FFPE tissues can be a valuable source in generating reproducible and biologically relevant proteomic profiles using reverse-phase protein arrays (RPPA).

We first evaluated a broad range of extraction conditions from FFPE tissues, including deparaffinization, rehydration, buffer components, pH conditions and temperatures. In the past, antigen retrieval techniques were used that mainly focused on applying high temperatures in appropriate buffers, facilitating the hydrolysis of formalin-induced crosslinks [19]. Subsequent studies hypothesized that the pH range may affect antigen retrieval and quality of immunostaining. In particular, the Tris–HCl buffer tended to produce better results at higher pH (pH = 8–9) [20]. Other authors have suggested that low pH around 4.0 can produce peptides resulting from acid-catalyzed aspartic acid cleavage, therefore lowering protein yield [21]. Addition of a reducing agent did not alter protein recovery significantly compared to pH [16]. Consistent with these studies, we observed that the highest protein yields were achieved using Tris–HCl containing 2% sodium dodecyl sulfate and 0.2 M glycine at pH 9.0 when heated at 100°C for 20 min, followed by incubation at 80°C for 2 h.

With respect to protein degradation, both Coomassie staining and western blot analysis revealed lower-weight products in the FFPE cell and xenograft samples, consistent with previous observations [5,22]. Since we initiated protein fixation immediately after harvesting, protein degradation may be the result of the duration of fixation, rather than solely due to handling prior to fixation. Our 16-hour fixation for cells and 24-hour fixation for xenografts may have led to challenges with isolating proteins from the fixed tissue, but this duration of fixation is commonly encountered in pathology laboratories. Novel extraction methods are continuously being developed to specifically address the duration of fixation in pathology laboratories, and it will be beneficial in the future, to adapt these protocols to being compatible with RPPA [23]. Besides the observed partial degradation, the quality of the signal was overall comparable to frozen extractions. This implies that partial protein degradation or protein modification may occur in FFPE samples, but not to the extent of significantly lowering protein yield or signal detection. This is an important point, since the downstream application, RPPA, represents a dot blot, and utilizes antibodies recognizing epitopes under denaturing conditions. This supports the notion that even partially degraded proteins will be readily detected using the RPPA approach, with little variation when compared to intact protein. Indeed, the dot blot approach of the RPPA may be even less influenced by protein degradation than the western blotting approach as RPPA only requires that the antibody epitope be intact and not the complete protein. This is supported by RPPA data which show that the average signal detected from the FFPE preparations was similar to that of the frozen counterparts (data not shown). In fact, the antibodies that exhibited marked difference between frozen and FFPE preparations across experiments (Table 2) did so without showing any bias towards a particular preparation. The observed differences in intensity for those markers between FFPE and frozen samples may be due to differential protein recovery rather than protein degradation, as suggested by previous mass spectrometry-based experiments [3]. This implies that some protein markers may perform better in FFPE compared to frozen or vice versa depending on the extent of protein recovery. Indeed, it may be appropriate to use other approaches such as clustering or correlations with clinical and other characteristics to define the “gold standard” rather than assuming that frozen samples represent the “truth”.

Previous studies have tested the applicability of RPPA on FFPE materials [3,24-27]. In this present study, we followed a systematic approach in evaluating feasibility, reproducibility and biological significance of RPPA applied on two distinct tissue types and for a wide range of protein markers. We first established a cell plug model, in order to eliminate experimental variability due to storage duration of FFPE tissue blocks, differences in fixing times, autolysis due to delayed fixing, individual differences in samples, anatomical or regional heterogeneity, and other possible sources of variability commonly occurring in human tissue FFPE preparations. The predictable patterns of protein phosphorylation according to pharmacological manipulations applied to cultured cells proved particularly useful in this study, in order to determine whether both FFPE and frozen preparations of treated cells were capable of reproducing the anticipated results. Changes in the AKT downstream effector phospho-S6 were inhibited or induced by inhibitors or growth factors to a similar degree in both FFPE and frozen cells. However, the increases induced by EGF in pAKT were not detected in FFPE samples suggesting degradation of pAKT. Nevertheless, the decrease in pAKT induced by LY294002 was faithfully captured in both frozen and FFPE preparations. A striking correlation of phosphorylation status between upstream activator and downstream effector was observed in tissues. In particular, factors in the same signaling pathway, AKT, 4EBP1 and S6, were coordinately phosphorylated in both FFPE and frozen preparations in the same patient samples interrogated (Figure 5A). Indeed, the correlation in patient samples was higher than that in the cell line samples. The usefulness of proteomic profiling of FFPE tissues in producing biologically meaningful information is further supported by the observation that global protein expression information from FFPE samples was capable of accurately categorizing renal tissues according to their underlying biology i.e. separating cancer from normal.

Using different types of tissues and cells in this study revealed a wealth of differences with respect to their proteomic profiles. As anticipated, protein markers performed markedly differently according to tissue type. Many of them were not detected according to tissue type, regardless of FFPE or frozen preparation status (Table 2). With respect to markers that demonstrated good correlation between FFPE and matched frozen samples, only 23 markers were common across all tissue sets (Figure 5A), as a testament to marked biological diversity of different cell lineages. This also suggests that it may be necessary to select different antibodies to interrogate the diverse array of tissue lineages as protein and particular phosphoprotein stability may be tissue specific.

With respect to the reproducibility of the approach, it is important to note that separate preparations, either FFPE or frozen, from the same tissue sample (replicates) performed similarly on RPPA (Figure 2). This questions the notion that tissue heterogeneity between replicate preparations may introduce critical variations in protein profiling preventing useful interpretation if laser-capture microdissection is not applied [28]. It is noteworthy that our replicate preparations were derived from adjacent serial sections or pieces, therefore limiting tumor heterogeneity. But unlike our replicate preparations from within the same sample, FFPE and matched frozen samples came from tissue pieces that were located a significant distance from one another, especially in the case of breast cancer samples. Thus, tissue heterogeneity may have contributed to discrepancies between FFPE and matched frozen RPPA results. This is consistent with recently published studies of spatial heterogeneity of DNA mutations [29].

With respect to the applicability of RPPA in the clinical routine setting, it is encouraging that the RPPA-based measurements of PR correlated significantly with the immunohistochemical results of this clinically significant marker in breast tumors (Figure 6).

FFPE tissues represent a valuable resource to conduct retrospective studies aimed to biomarker discovery and validation, in cancer as well as other diseases. The most effective approach may be to both discover and validate molecular markers on FFPE preparations to decrease variability potentially induced by tissue handling when comparing FFPE and frozen samples. In order to improve the ability to characterize proteins from tissues used for pathological evaluation, a number of steps could be considered: using a consistent and short period of fixation in formalin, limiting the duration of time between tissue collection and addition of formalin, using small tissue pieces where formalin will permeate more frequently, obtaining tissue from multiple parts of the tissue and the consideration of new fixation approaches that are under development. Clearly, FFPE tissue studies hold the promise of producing highly reproducible and meaningful data when linked to powerful high-throughput methodologies such as reverse-phase protein arrays.

## Materials and methods

### Preparation of FFPE cell blocks

MDA-MB-231 and MDA-MB-468 cells were plated in 15 cm Petri dishes, and allowed to grow to 80% confluence. They were subsequently starved with serum-free medium overnight and then incubated with EGF or IGF for 10 minutes for cell signaling stimulation. The starved and growth factor stimulated cell monolayers were rinsed twice with TBS (50 mM Tris–HCl pH 7.5 and 150 mM NaCl) and harvested with rubber cell scrapers. Then cell pellets were processed as fresh frozen or FFPE cell blocks. Cell pellets were fixed in 10% formalin (Fisher Scientific, Pittsburgh, PA, USA) for 16 hours, and embedded in paraffin as a piece of tissue according to standard histological procedures. Paraffin cell blocks were stored at room temperature until analysis.

### Xenografts and human tissues

The mammary pads of four-week-old female nude mice were inoculated with 5 × 10^6^ MDA-MB-231 cells. Mice with mean tumor diameters of 0.5 cm were sacrificed and the grafted tumors were cut in halves. Half tumor was fixed in 10% formalin for 24 hours and paraffin embedded using standard procedures; the other half was immediately snap-frozen and then stored at −80°C until use. All experiments with mice were performed according to the IACUC guidelines for the care and use of living animals in scientific research.

Renal tumor, renal cortex and breast tumor tissue FFPE blocks and their matched frozen pieces from the same patients were obtained from the University of Texas MD Anderson Cancer Center tissue bank, following Institutional Review Board (IRB) approval and guidelines. In the case of renal tissues, the FFPE and matched frozen preparations were derived from adjacent tissue pieces, whereas in the case of breast cancer tissues, they were derived from distant tumor pieces. Renal tissue samples were confirmed to have at least 70% cellular content, whereas the breast cancer tissue samples had at least 50% cellular content. All samples used in the study were histologically confirmed using H&E.

### Deparaffinization and protein extraction

To define technical reproducibility of methodology, replicates of four serial FFPE cell or tissue sections, 10 μm thick, were placed in Eppendorf tubes and deparaffinized by incubation at room temperature in xylene (Fisher Scientific, Pittsburgh, PA, USA) for 10 min. After each incubation, tissue was pelleted at 14000 × g for 3 min, and incubation/centrifugation steps were repeated two times. The deparaffinized tissue pellets were then rehydrated with a graded series of ethanol (Pharmaco products Inc, Brookfield, IL, USA). The rehydrated tumor tissue and cell sections were resuspended in a panel of extraction buffers as described (see Table 1). The extraction buffers evaluated included Qproteome FFPE tissue kit (Qiagen); 20 mM Tris HCl buffers at various pH values (4, 6 and 9), with 2%(w/v)SDS; the lysis buffer routinely used for RPPA assay (1% Triton X-100, 50 mM Hepes, pH 7.4, 150 mM NaCl, 1.5 mM MgCl_2_, 1 mM EGTA, 10 mM NaF, 100 mM Na Pyruvate, 1 mM Na_3_VO_4_, 10% glycerol, containing protease inhibitors and phosphatase inhibitors from Roche Applied Science) [9] plus 4 × SDS buffer (3:1, v/v). For tissue samples, the amount of extraction buffer added to the pellets from paraffin sections was estimated according to the size of the tissue surface area. In particular, tissue blocks were first scanned with an Image Scanner (CanoScan 8400F, Canon, Lake Success, NY, USA) and then the surface area of the paraffin-embedded tissue was calculated using ImageJ (NIH). For frozen samples, adjustment of buffer volume according to weight of each frozen piece was performed.

The samples were first incubated on ice for 5 min, and mixed by vortexing, then boiled at 100°C for 20 min followed by an optional sonication step and an optional incubation at 80° C in a water bath for 2 hours. The extraction methods followed are described in detail in Table 1. After protein extraction, any remaining unsolubilized material was pelleted at 14000 × g for 20 min, and protein concentration of total protein extracted was determined by the BCA Protein Assay (Pierce Chemicals Co., Rockford, IL, USA). The Pierce BCA Protein Assay is a detergent-compatible formulation and the protein standards were prepared using the same lysis buffer as the samples.

### SDS-PAGE and western blot

Protein extracts obtained from fresh-frozen and FFPE tissues were subjected to SDS-PAGE in a Bio-Rad Mini Protean II system. Proteins were stained with Brilliant R 250 according to Westermeier *et al.*[30]. Parallel gels were transferred onto PVDF (Polyvinylidene Fluoride, BioRad, Richmond, USA) membrane. Membranes were blocked and probed with antibodies against different sized molecules and visualized using ECL (Amersham/GE health care).

### RPPA assays and analysis

Protein extracts from cell and tissue paraffin blocks were probed for expression of validated antibodies by RPPA [9,11,31,32]. Specifically, five serial 2-fold dilutions of the protein extracts were performed using RPPA lysis buffer containing 1% SDS. The diluted lysates were spotted on nitrocellulose-coated FAST slides (Whatman, Schleicher & Schuell BioScience, Inc., Keene, NH) by an Aushon 2470 arrayer (Aushon Biosystems, Burlington, MA) per manufacturer’s protocol. The construction of the protein array is shown in Additional file 5: Figure S1. Each spotted slide was incubated with a primary antibody (Additional file 1: Table S1) in the appropriate dilution. A total of 169 slides were stained for 169 antibodies. The specific protein-antibody interaction was recognized by biotin-conjugated secondary antibody and amplified by tyramide deposition. The analyte was detected by avidin-conjugated peroxidase reactive to its substrate chromogen diaminobenzidine (DAKO catalyzed signal amplification (CSA) system, DAKO, Carpinteria, CA). The stained RPPA slides were scanned by the Hewlett-Packard (HP) scanner and its companying scanning software, and the slide images were quantified for raw signal intensities by the software MicroVigene (VigeneTech Inc., Carlisle, MA) per manufacturer’s protocol. The raw signal intensities (which are provided in detail, in Additional files 2, 3 and 4: Tables S2, S3, and S4) were then processed by the R package SuperCurve [33] developed by the Department of Bioinformatics and Computational Biology at University of Texas MD Anderson Cancer Center ( http://bioinformatics.mdanderson.org/Software/OOMPA/). The log2-scaled protein concentrations were normalized by global sample median normalization (GSMN) (the median of all protein marker intensities for a single sample is subtracted from each of the data points of this specific sample [22,34]). Student’s *t* test was used to evaluate statistical significance of differences in protein expression levels between FFPE and frozen sample groups, or between PR-negative and PR-positive breast cancer groups, in order to determine markers with intensity differences between FFPE and frozen preparations, or between PR-positive and PR-negative tumors. The reproducibility of sampling methods was determined by Spearman correlation coefficient. Principal component analysis was performed to show the relatedness of the samples and of the proteins. All statistical analysis was performed using the statistics software package R [35].

## Abbreviations

BCA: Bicinchoninic Acid; ECL: Enhanced Chemiluminescence; FF: Fresh Frozen; FFPE: Formalin-fixed Paraffin-embedded; GSMN: Global Sample Median Normalization; PVDF: Polyvinylidene Fluoride; RPPA: Reverse-phase Protein Array; SDS-PAGE: Sodium Dodecyl Sulfate Polyacrylamide Gel Electrophoresis.

## Competing interests

The authors declare that they have no competing interests.

## Authors’ contributions

HG: experiments and manuscript preparation, WL: statistics, ZJ: statistics, PT: tissue evaluation/pathology, EJ: tissue evaluation, GBM: manuscript preparation, YL: RPPA experiments and manuscript preparation, BTH: manuscript preparation, DT: RPPA experiments and manuscript preparation. All authors read and approved the final manuscript.

## Authors’ information

HG: Research scientist, Systems Biology, MD Anderson Cancer Center, WL: Research Statistical Analyst, MD Anderson Cancer Center, ZJ: Sr. Statistical Analyst, MD Anderson Cancer Center, PT: Associate Professor, Pathology, MD Anderson Cancer Center, EJ: Associate Professor, Genitourinary Medical Oncology, MD Anderson Cancer Center, GBM: Professor, Chair, Systems Biology, MD Anderson Cancer Center, YL: Associate professor, Systems Biology, director of RPPA Core, BTH: Senior Lecturer, RCSI, Consultant Medical Oncologist, Beaumont Hospital, Royal, College of Surgeons of Ireland, Dublin, Ireland, DT: Instructor, Genitourinary Medical Oncology, MD Anderson Cancer Center.

## Supplementary Material

Additional file 1 **Table S1.** List of the primary antibodies used in the RPPA experiments. Click here for file

Additional file 2 **Table S2.** RPPA raw signal intensities of the cell lines experiment (FFPE and frozen). Click here for file

Additional file 3 **Table S3.** RPPA raw signal intensities of the breast cancer tissue experiment (FFPE and frozen). Click here for file

Additional file 4 **Table S4.** RPPA raw signal intensities of the renal tissue experiment (FFPE and frozen). Click here for file

Additional file 5 **Figure S1.** Protein Microarray Construction. The size, design and dilution arrangement of the protein microarray are shown. Click here for file

Additional file 6 **Figure S2.** SDS-PAGE analysis of protein lysates extracted from fresh frozen and FFPE cell blocks or xenografts by using different extraction protocols. (A) Extracts from breast cancer cell lines incubated with or without EGF were derived from FFPE or fresh preparations using different extraction methods and subjected to SDS-PAGE analysis. (B) SDS-PAGE image of protein extracts from fresh frozen and FFPE xenograft tissues are shown. Lane M: molecular weight marker. Arrows point to low molecular weight bands attributed to protein degradation. Click here for file
