# Supplementary material for: An efficient procedure for protein extraction from formalin-fixed, paraffin-embedded tissues for reverse phase protein arrays
Source: Proteome Sci. 2012 Sep 24;10:56. doi: 10.1186/1477-5956-10-56 (PMC3561137; doi:10.1186/1477-5956-10-56)
Supplement: Additional file 5 — Figure S1. Protein Microarray Construction. The size, design and dilution arrangement of the protein microarray are shown. [file 1477-5956-10-56-S5.ppt]

## Slide 1
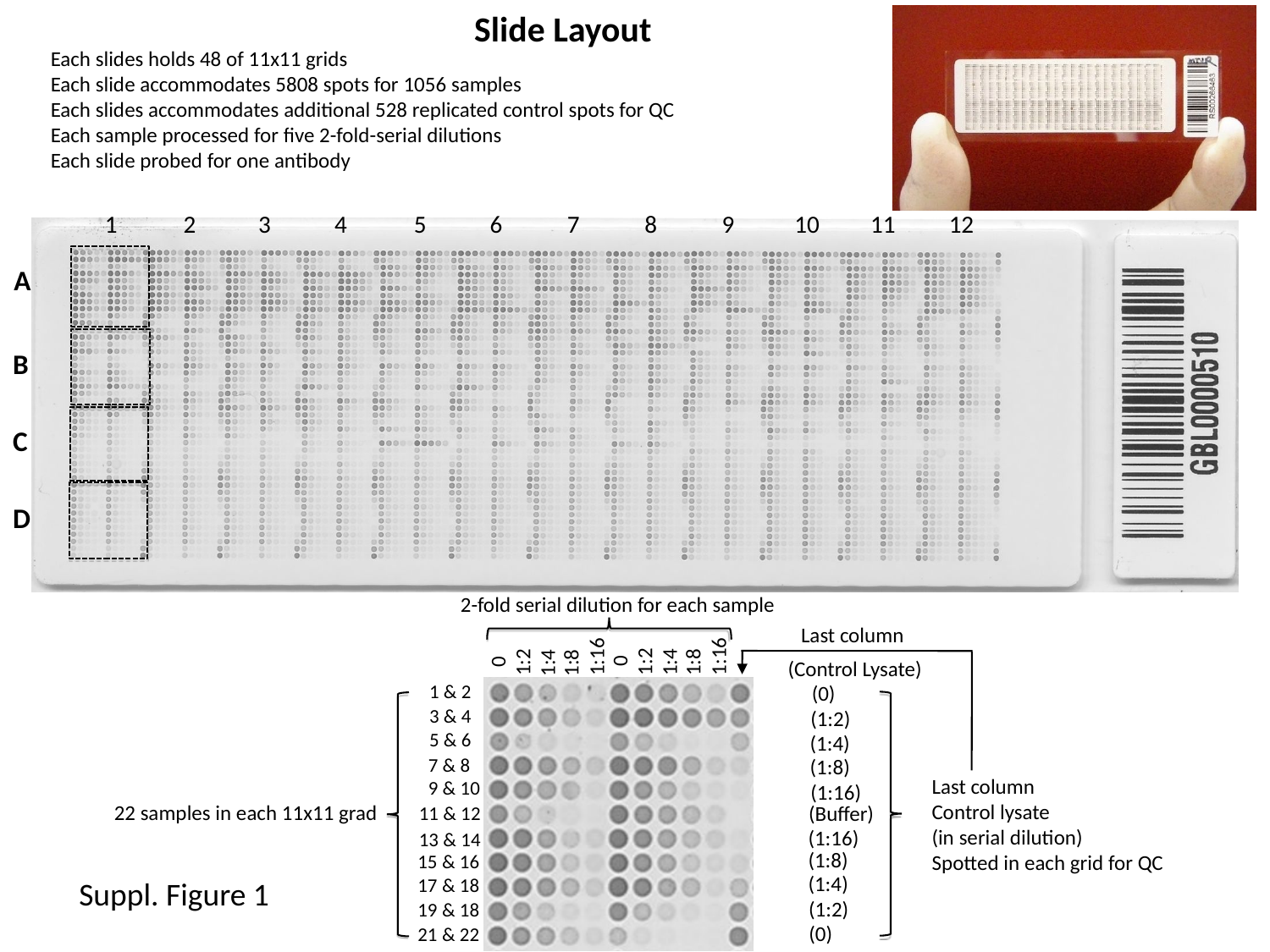

Slide Layout
Each slides holds 48 of 11x11 grids
Each slide accommodates 5808 spots for 1056 samples
Each slides accommodates additional 528 replicated control spots for QC
Each sample processed for five 2-fold-serial dilutions
Each slide probed for one antibody
1
2
3
4
5
6
7
8
9
10
11
12
A
B
C
D
2-fold serial dilution for each sample
1:16
1:2
1:8
0
1:4
1:16
1:2
1:8
0
1:4
Last column
(Control Lysate)
1 & 2
3 & 4
5 & 6
7 & 8
9 & 10
11 & 12
13 & 14
15 & 16
17 & 18
19 & 18
21 & 22
(0)
(1:2)
(1:4)
(1:8)
(1:16)
(Buffer)
(1:16)
(1:8)
(1:4)
(1:2)
(0)
Last column
Control lysate
(in serial dilution)
Spotted in each grid for QC
22 samples in each 11x11 grad
Suppl. Figure 1
