# Supplementary figures and images for: An efficient procedure for protein extraction from formalin-fixed, paraffin-embedded tissues for reverse phase protein arrays
Source: Proteome Sci. 2012 Sep 24;10:56. doi: 10.1186/1477-5956-10-56 (PMC3561137; doi:10.1186/1477-5956-10-56)

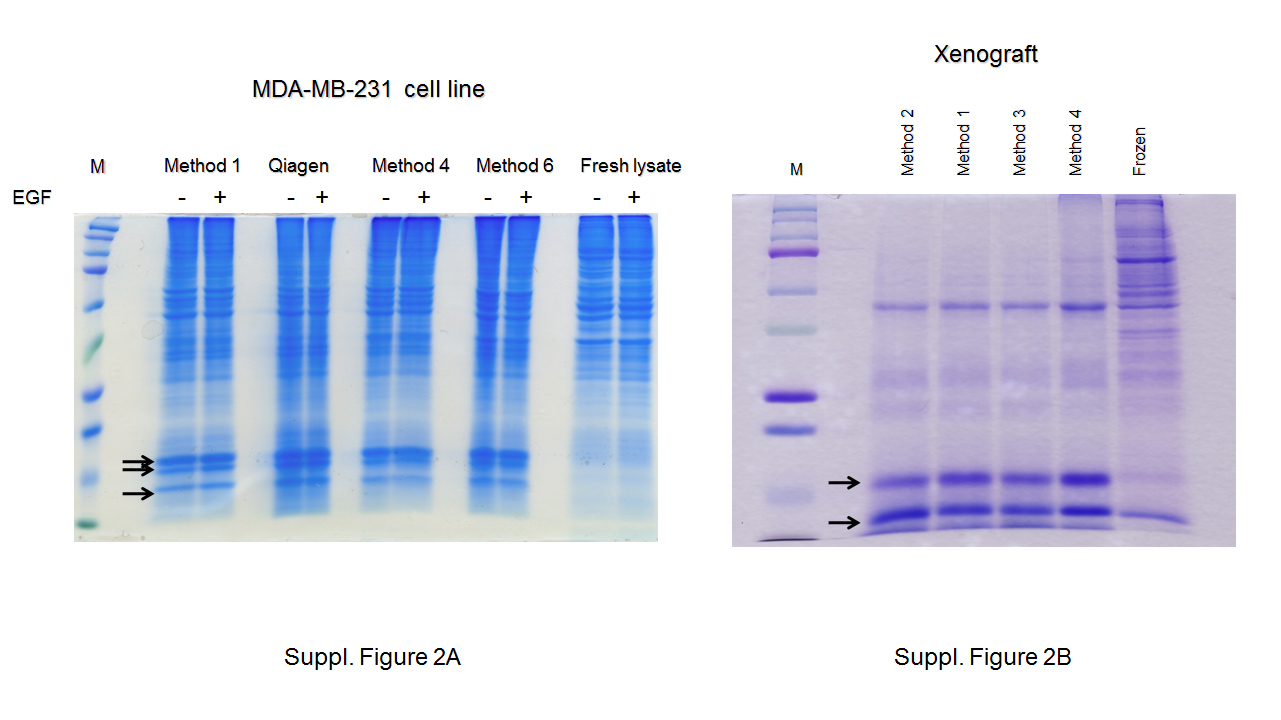

Supplement: Additional file 6 — Figure S2. SDS-PAGE analysis of protein lysates extracted from fresh frozen and FFPE cell blocks or xenografts by using different extraction protocols. (A) Extracts from breast cancer cell lines incubated with or without EGF were derived from FFPE or fresh preparations using different extraction methods and subjected to SDS-PAGE analysis. (B) SDS-PAGE image of protein extracts from fresh frozen and FFPE xenograft tissues are shown. Lane M: molecular weight marker. Arrows point to low molecular weight bands attributed to protein degradation. [file 1477-5956-10-56-S6.jpeg]
